# Supplementary material for: Why health system diagnosis delay among tuberculosis patients in Illubabor, Oromia region, South West Ethiopia? A qualitative study
Source: PLoS One. 2022 Dec 30;17(12):e0278592. doi: 10.1371/journal.pone.0278592 (PMC9803213; doi:10.1371/journal.pone.0278592)
Supplement: S1 Checklist — (PDF) [file pone.0278592.s001.pdf]

A Checklist for availability of infrastructure and other resources

Instruction: This checklist should be completed by observing the health facilities and asking persons in charge. The presence and absence should be verified through observation.

Permission for observation and interview

Dear Sir/Madam My name is --------------------------------------. I am a member of a study team conducting research on Tuberculosis diagnosis delay in Illubabor Zone. The aim of this study is to assess the over performance and quality of TB DOTS strategy. Then to provide important information which will help for program improvement. This study's findings will assist us in providing helpful information to decision makers so that program performance and quality may be enhanced and community members can receive better services. This study was chosen at random for your district. I'll ask you to give me some program resource information. The information you supply will be handled directly and confidentially and will not be shared with anybody who knows your name or who will identify you as the source. I'd like to request your permission to observe the infrastructure and other resources associated with the TB control program. The data gathered during this observation will also be kept totally confidential. The research is approved by the Research and community Service Coordination Office (RCS) of Mettu University Faculty of Public Health and Medical Sciences.

Can I get your cooperation in providing necessary information and documents?

1. Yes ---------- 2. No ------------

Name and signature of data collector _____________________________ Date ________________

Name and signature of data supervisor ____________________________ Date ________________

DOTS site/ Health office --

District---

Types of health facility--

**Over all infrastructure and patient environment**	Yes	No	NA	Remark
Does the facility have trained laboratory personnel? (on AFB techniques)				
Is there waiting area for patient?				
Does the facility have separate and equipped room for TB clinic?				
Does the health facility have safe water supply?				
Does the health facility have electricity?				
Is appropriate sign posted to locate TB clinic?				
**Over all infrastructure and patient environment …**				
Are there information education and Communication (IEC) materials posted at patient waiting area and OPD room at visible place in local language				
**Outpatient departments (OPDs)for availability of the following**				
National TB control program guideline				
Laboratory AFB request paper				
Drug prescription paper				

Usable OPD abstract				
**Laboratory unit for availability of the following**				
National TB control program guideline				
Standard operating procedures of AFB (SOPs)				
Laboratory AFB register				
Carbon fuchsine				
Acid alcohol				
Methylene blue				
Availability of functional X ray				
Functional Microscope (Write type of microscope as remark)				
Alarm clock				
Staining rack				
Drying rack				
Spirit lamp				
Forceps				
Slides (Write type of slides as remark)				
Sputum cups				
Immersion oil				
Lens tissue				
Disinfectant (either 5% phenol or 10% sodium hypo chloride)				
Filter paper				
Applicator stick /wire loop				
Glove				
**TB clinic (check for expiry date for the drugs)**				
Is TB consultation room clean?				
Is there adequate light in TB room?				
Is there adequate ventilation in TB room?				
Are there IEC materials with local language posted at visible spaces?				
Is there NTCP manual?				
Is there reporting formats				
Is there functional standard unit register?				
Is there functional weighting scale?				
Does TB room provide privacy?				
Is there Masks				
Is there appropriate safety box				
**Pharmacy Store for availability of the following** (check for expiry date for the reagents and drugs)				

Sputum cup				
Slides				
Carbol fuchsine				
Methylene blue				
Acid alcohol				
Immersion oil				
Applicator stick				
**Review stock card for the following if stock out for the last three months**				
Sputum cup				
Slides				
Carbol fuchsine				
Methylene blue				
Acid alcohol				
Immersion oil				
Applicator stick				
**Check availability of stock for at least the next two months**				
Sputum cup				
Slides				
Carbol fuchsine				
Methylene blue				
Acid alcohol				

NA* - Not applicable

Description of Health facility /DOTS site and rooms

Other interesting observations (environment)
