# Supplementary material for: Why health system diagnosis delay among tuberculosis patients in Illubabor, Oromia region, South West Ethiopia? A qualitative study
Source: PLoS One. 2022 Dec 30;17(12):e0278592. doi: 10.1371/journal.pone.0278592 (PMC9803213; doi:10.1371/journal.pone.0278592)
Supplement: S2 Checklist — (DOCX) [file pone.0278592.s002.docx]

COREQ (Consolidated criteria for Reporting Qualitative research) checklist

| **Topic** | **Item. No.** | **Guide Questions/Description** | **Reported on** |
| --- | --- | --- | --- |
| **Domain 1: Research team and reﬂexivity** | | | |
| ***Personal characteristics*** | | | |
| Interviewer/facilitator | 1 | Which author/s conducted the interview or focus group? | Page 7, method and materials section,  Data collection method and tool sub  Section |
| Credentials | 2 | What were the researcher’s credentials? E.g. PhD, MD | Page 7, method and materials section,  Data collection method and tool sub  Section |
| Occupation | 3 | What was their occupation at the time of the study? | Page 7, method and materials section,  Data collection method and tool sub  Section |
| Gender | 4 | Was the researcher male or female? | Page 7, method and materials section,  Data collection method and tool sub  Section |
| Experience and training | 5 | What experience or training did the researcher have? | NA |
| ***Relationship with participants*** | | | |
| Relationship established | 6 | Was a relationship established prior to study commencement? | Page 7, method and materials section,  Data collection method and tool sub  Section |
| Participant knowledge of  the interviewer | 7 | What did the participants know about the researcher? e.g. personal, goals, reasons for doing the research | Page 9 & 10, method and materials section Ethical considerations sub section. |
| Interviewer characteristics | 8 | What characteristics were reported about the interviewer/facilitator?  e.g. Bias, assumptions, reasons and interests in the research  topic | Page 8&9, method and materials section,  Data analysis, data quality control and trustworthiness sub  Section |
| **Domain 2:Study design** | | | |
| *Theoretical* *framework* | | | |
| Topic | Item No. | Guide Questions/Description | Reported on |
| Methodological orientation and Theory | 9 | What methodological orientation was stated to underpin the study? e.g. grounded theory, discourse analysis, ethnography, phenomenology, content analysis | Page 8&9, method and materials section,  Data analysis, data quality control and trustworthiness sub  Section |
| *Participant selection* | | | |
| Sampling | 10 | How were participants selected? e.g. purposive, convenience, consecutive, snowball | Page 6&7,method and materials section Sample size and sampling techniques subsection |
| Method of approach | 11 | How were participants approached? e.g. face-to-face, telephone, mail, email | Page 7&8,method and materials section Data collection method and tool subsection |
| Sample size | 12 | How many participants were in the study? | Page 6&7,method and materials section Sample size and sampling techniques subsection |
| Non-participation | 13 | How many people refused to participate or dropped out? Reasons? | N/A |
| Setting | | | |
| Setting of data collection | 14 | Where was the data collected? e.g. home, clinic, workplace | Page 7, method and materials section Data collection method and tool subsections |
| Presence of non-participants | 15 | Was anyone else present besides the participants and researchers? | Page 7, method and materials section Data collection method and tool subsections |
| Description of sample | 16 | What are the important characteristics of the sample? e.g. demographic data, date | Page 11, result section, table 1 |
| *Data collection* | | | |
| Interview guide | 17 | Were questions, prompts, guides provided by the authors?  Was it pilot tested? | Page 7, method and materials section,  Data collection method and tool sub  Section |
| Repeat interviews | 18 | Were repeats interviews carried out? If yes, how many? | N/A |
| Audio/visual recording | 19 | Did the research use audio or visual recording to collect the data? | Page 7&8,method and materials section Data collection method and tool subsection |
| **Topic** | **Item**  **No.** | **Guide Questions/Description** | **Reported on** |
| Field notes | 20 | Were ﬁeld notes made during and/or after the interview or focus group? | Page 7&8,method and materials section Data collection method and tool subsection |
| Duration | 21 | What was the duration of the inter views or focus group? | Page 8,method and materials section Data collection method and tool subsection |
| Data saturation | 22 | Was data saturation discussed? | Page 7,method and materials section Data collection method and tool subsection |
| Transcripts returned | 23 | Were transcripts returned to participants for comment and/or Correction? | No, but peer debriefing done by listing the de-identified audio taped interviews and checking with the transcripts, Page 8&9, method and materials section Data analysis, data quality control and trustworthiness sub section |
| **Domain 3: analysis and ﬁndings** | | | |
| *Data analysis* | | | |
| Number of data coders | 24 | How many data coders coded the data? | Page 8&9, method and materials section Data analysis, data quality control and trustworthiness sub section |
| Description of the coding tree | 25 | Did authors provide a description of the coding tree? | N/A |
| Derivation of themes | 26 | Were themes identiﬁed in advance or derived from the data? | Page 9, method and materials section Data analysis, data quality control and trustworthiness sub section |
| Software | 27 | What software, if applicable, was used to manage the data? | N/A |
| Participant checking | 28 | Did participants provide feedback on the ﬁndings? | N/A |
| **Topic** | **Item**  **No.** | **Guide Questions/Description** | **Reported on** |
| *Reporting* | | | |
| Quotations presented | 29 | Were participant quotations presented to illustrate the themes/ﬁndings?  Was each quotation identiﬁed? e.g. participant number | Page 11- 19, result section |
| Data and ﬁndings consistent | 30 | Was there consistency between the data presented and the ﬁndings? | Page 14,15,16 & 18, result section |
| Clarity of major themes | 31 | Were major themes clearly presented in the ﬁndings? | Page 11- 19, result section |
| Clarity of minor themes | 32 | Is there a description of diverse cases or discussion of minor  themes? | Page 11- 19, result section |
