# Supplementary material for: Why health system diagnosis delay among tuberculosis patients in Illubabor, Oromia region, South West Ethiopia? A qualitative study
Source: PLoS One. 2022 Dec 30;17(12):e0278592. doi: 10.1371/journal.pone.0278592 (PMC9803213; doi:10.1371/journal.pone.0278592)
Supplement: S1 Questionnaire — (PDF) [file pone.0278592.s003.pdf]

1. Interview guide for Zonal /district TB program coordinator/supervisors, DOT providers and laboratory professionals.

Background information

Sex ------------- Age -------------------- Educational level-------------------------------

Experience ---

Introductory/open questions:

1. Could you please tell me about your experience with the national tuberculosis control program?

2. Could you please tell me what resources or components of the treatment program are required to avoid TB diagnosis delays?

3. Do you believe this Zone/District has the infrastructure and other resources necessary to avoid TB diagnosis delays at health facilities?

Probe: If not, what resource constraints in your Zone/districts could be cited as a cause of diagnosis delays?

What could be the reasons for the lack of sufficient resources?

4. Has there been a service outage owing to a lack of resources?

Probe: If so, what resources are you referring to?

What steps were made to resolve the problem?

5. What are your thoughts on the process/activities used by health care practitioners in this Zone/district to delay diagnosis?

Probing: Have you carried out any supportive supervision' this year? How often do you do it? Was feedback given in writing or orally?

6. How do you think health professionals are adhering to national TB prevention and control

guidelines? **Probe**: During diagnosis

7. Do you believe that the knowledge and skill of health professionals is sufficient for the diagnosis of tuberculosis?

Probe: If no, why?

8. Do they have any TB diagnostic training?

9. Do you have any other information or comments about TB diagnosis that you'd like to share?

If yes, please describe how it is related to the health-care system's delay.

I. Interview guide for TB patients on DOT.

Background information

Sex ------------- Age -------------------- Residence---

Types of TB-------------------------------

Duration of treatment --

Introductory/open questions:

1. Could you please tell me about your experience health service related to Tuberculosis?

2. Do you believe this Zone/District has the infrastructure and other resources necessary to avoid TB diagnosis delays at health facilities?

Probe: If not, what resource constraints in these Zone/districts could be cited as a cause of diagnosis delays?

What could be the reasons for the lack of sufficient resources?

3. Has there been a service outage owing to a lack of resources?

Probe: If so, what resources are you referring to?

4. What are your thoughts on the approaches used by health care practitioners during diagnosis?

5. Do you have any other information or comments about TB diagnosis that you'd like to share?

If yes, please describe how it is related to the health-care system's delay.
