# Supplementary material for: Why health system diagnosis delay among tuberculosis patients in Illubabor, Oromia region, South West Ethiopia? A qualitative study
Source: PLoS One. 2022 Dec 30;17(12):e0278592. doi: 10.1371/journal.pone.0278592 (PMC9803213; doi:10.1371/journal.pone.0278592)
Supplement: Attachment — Submitted filename: Response to Reviewers.docx [file pone.0278592.s005.docx]

**Response to Reviewers**

Reviewer #1: Overall comments;
It is an important article that explains qualitative aspects of delay in diagnosis of TB in low middle income countries. The authors have revised it significantly. However, I still feel the English language and expression needs improvement at multiple points. There are multiple typo errors: in some places there is a space between bracket and figures and in certain places without a space, which needs to be corrected. Please add the revision of methods suggested by the reviewers in the manuscript.

Response: Thank you. We have modified accordingly.
Abstract:
Comment No 1:
Background: “The main target of Tuberculosis control and prevention is to detect incident cases as early as possible”. However, the target is hard to meet due to problems that exist within health facilities, which lead to diagnostic delays. Despite this, there is no information explored why diagnosis delays among Tuberculosis patients.” This statement is not correct. The main target of TB control and prevention is not only to detect incident cases as quickly as possible but also to prevent occurrence of disease. This is also the responsibility of the health facility to screen the contacts, identify cases and prevent spread in children by identifying children for prophylactic therapy.

Response: Yes, it has been corrected accordingly. see first paragraph on page 2.

Some examples of the problems stated above are;
Comment No 2
Results: Diagnosis delays have been identified as a result of issues with sample collection procedures, poor competency of health professionals, absences/scarcity of health professionals, and scarcity of medical products and absence/scarcity of basic infrastructure. This sentence can be rephrased to avoid repetition of absence/ scarcity.

Response: Yes, it has been rephrased accordingly. See the result part of abstract.
Comment No 3; Result: “We found 18 health facilities without skilled personnel in the OPD, seven health facilities with a broken microscope, and almost all health facilities without a
separate room for sputum examination”. Grammatical correction needs to be done 7 health facilities had a broken microscope?

Response: Yes, it has been rephrased accordingly. See the result part of abstract.
Comment no 4: Conclusion: Many reasons for TB diagnosis delays have been identified in the study area. Poor competence of health workers and scarcity of resources were identified. Depending on the finding, we suggest strengthening the health workers’ training. Concrete strategies need to be designed to retain Professionals. Training on human resource planning and budget preparation is needed for low level managers. The meaning of low level managers is not clear.

Response: Low level managers mean frontline managers. Now we have replaced with frontline managers. Check the last line of conclusion in the Abstract
Introduction:
Comment No 5: Introduction line 3; why expected 10.4 million cases? When the year is already complete and should have the actual figures. Also, why has the statistics of 2016 been mentioned instead of the latest? Please put the latest statistics.

Response: we have updated the information. check second page, line three
Comment No 6; “The time elapsed between seeking health care and receiving a diagnosis is defined as diagnosis delay” Why is definition in the past tense in introduction?
Response: We have modified accordingly. Check second paragraph, line one under the introduction.
Comment No 7; “Diagnosis of tuberculosis cases has been identified as a key impediment
to TB control, particularly in low-income countries such as Ethiopia”. Probably the author wants to state that early diagnosis or prompt diagnosis is a key impediment to ......
Response: You are right. We have corrected accordingly.
Comment No 8; “Because, it causes more acute sickness, a longer period of infection, and worse treatment outcomes such as mortality and drug resistance”. Please replace with it causes severe acute phase of the disease.

Response: Yes, we have replaced accordingly. check second paragraph, fourth line under the introduction.
Comment No 9:
Furthermore, despite "free service" for TB, it results in TB patients suffering significant pre-diagnosis costs for care seeking. Add s to service.
Response: Good. it is corrected.
Comment No 10:
According to the findings of a systematic review and meta-analysis, diagnosis delays ranged
from 2 to 128.5 days, whereas they ranged from 6 to 28 days in other studies conducted in
Ethiopia among tuberculosis patients (10, 11). Is 2 or 6 days counted as diagnostic delay or is it normal time taken to diagnose TB????

Response: Actually the ranges are mentioned to show the existing days of delay from the literature. But different literatures utilized the median as cut point.
