# Supplementary material for: Why health system diagnosis delay among tuberculosis patients in Illubabor, Oromia region, South West Ethiopia? A qualitative study
Source: PLoS One. 2022 Dec 30;17(12):e0278592. doi: 10.1371/journal.pone.0278592 (PMC9803213; doi:10.1371/journal.pone.0278592)
Supplement: Attachment — Submitted filename: Response to Reviewer.docx [file pone.0278592.s006.docx]

**Response to Reviewer**

1. The article needs to be read and revised by a native English speaker.

Response: Yes, I have invited the English language expert to read and revise the manuscript. Consequently, the necessary improvement has been made.

2. The title of the article needs to be revised/rewritten

Response: Of course, revised. See the track change

3. Reviewer comment: In the introduction section and throughout the manuscript, definition of health system delay, ‘’Early diagnosis and initiation of tuberculosis treatment’’ ‘’Late detection and Treatment’’ are not clear.

Response: see the second paragraph of track change under the introduction part.

4. Methods and Materials

Study setting and period

Study period has been written in the reverse order ‘’from October 15, 2021, to March 1, 2021’’.

Response: We have corrected the order accordingly (see study setting and period subsection).

5. Sample size and sampling technique

In-depth interview

It is not mentioned that 28 health professionals were chosen out of how many health professionals?

Response: Actually, Illubabor Zone has 13 districts and one town administration. Out of these, six districts and one town were selected randomly. As mentioned in the manuscript, the health professionals involved were program managers, laboratory professionals, and DOT providers. Regarding the six program managers, one was from the Zonal CDC focal person and the other five were from selected district health offices. Regarding the DOT providers, 17 were included out of 82 DOT providers in the seven districts. Five of 36 laboratory experts were from seven health facilities.

6. Why was the criteria of 6 months of experience in TB for health professionals chosen?

Response: After six months, we expect any health professional, know their work environment. They can give enough information about the reasons for the TB diagnosis delay.

7. What was the criteria for choosing different numbers of interviewees(Seventeen DOT providers, five laboratory experts, six program)

Response: For triangulation, different numbers of health professionals were interviewed.

8. Facility Observation

Who observed, what was their qualification, how frequently for how long they observed the facilities?

Response: At each facility, observations were conducted for 50 to 70 minutes by the authors. They had masters in educational qualifications (see 124 & 125 lines from the track change version).

9. Interviewers

What was their qualification, were they trained in interviews and were their methods checked?

Response: The interviewers were the authors (originators of the research idea).We had a common understanding of data collection procedures.

10. Finally until the publication was approved? ??? the authors had regular conversations and consultations about the design, data analysis, and result interpretations. Unclear and unnecessary information is given.

Response: We have removed it. See track change version.

11. Ethics approval and consent to participate

Were the participants compensated for their time?

Was any incentive given to the study participants?

Response: Any incentives were not given to participants.

12. The sample collection method is not clear regarding different collection procedures such as ‘’spot spot’’ and ‘’spot morning spot’’ techniques.

Response: A spot morning spot technique is for sputum microscopy. One spot sample is collected at the time of the first visit of a patient to the laboratory. Two sputum (spot and early morning) samples were collected the next day.

In 2011, WHO advice was revised with a recommendation of a two-‘spot-spot’ strategy collected on the same day. According to the participants, after the TB diagnosis technique changed from spot morning spot to spot-spot it has been contributing to the diagnosis delay.

13. A criteria of trained professional in OPD has not been mentioned.

Response: Any health professional who has taken any type of in-service TB training is considered a trained health professional.

14. Discussion
    First paragraph, No new finding has been reported as a result of the study. Scarcity of infrastructure is already known.

Response: OK, This research topic is new. Because previous studies were conducted quantitatively and others were conducted on factors affecting case identification. But, this research explored reasons for diagnosis delays qualitatively. In particular, it is helpful for the study area. Because contributing factors differ across societies, types of health facilities visited, and geographical regions between population groups in the same local settings and disease categories. Localized studies are needed to uncover area and population-specific characteristics linked to extended TB diagnosis delays.

15. Were the skills of the lab professionals checked?

Response: Dear Reviewer, The study's intention is not to measure (check) the skills of lab professionals. We have just recorded factors delaying TB diagnosis by participants.

16. Conclusion is too long in abstract and end of discussion. Needs to be rewritten.

Response: Thank you. It has been modified accordingly; see unmarked version.
